# 肿瘤突变负荷对肺癌免疫治疗疗效的预测价值

**DOI:** 10.3779/j.issn.1009-3419.2019.06.08

**Published:** 2019-06-20

**Authors:** 焕兰 撒, 克威 马, 勇 高, 德强 王

**Affiliations:** 1 256603 滨州，滨州医学院附属医院疼痛科 Department of Pain Management, Affiliated Hospital of Binzhou Medical University, Binzhou 256603, China; 2 130021 长春，吉林大学第一医院肿瘤中心 Cancer Center, the First Hospital of Jilin University, Changchun 130021, China

**Keywords:** 免疫治疗, 免疫检查点抑制剂, 肺肿瘤, 肿瘤突变负荷, Immunity therapy, Immunological checkpoint inhibitors, Lung neoplasms, Tumor mutation burden

## Abstract

肺癌的发病率和死亡率居全球首位，除化疗、放疗和靶向治疗外，免疫治疗也成为其不容忽视的治疗策略。美国食品药品监督管理局（Food and Drug Administration, FDA）相继批准免疫检查点抑制剂作为晚期非小细胞肺癌（non-small cell lung cancer, NSCLC）二线治疗和部分患者一线治疗的标准方案。美国国家综合癌症网（National Comprehensive Cancer Network, NCCN）也推荐免疫检查点抑制剂用于复发小细胞肺癌（small cell lung cancer, SCLC）的治疗。目前肺癌治疗已进入精准治疗时代，因此挑选有效及可靠的生物标记物，筛选出接受免疫治疗的优势人群成为精准治疗的重点。越来越多的研究提示肿瘤突变负荷（tumor mutation burden, TMB）未来可能成为免疫治疗的独立预测生物标记物，但是TMB在预测免疫疗效方面仍有一定局限性。本文综述了TMB对于肺癌免疫治疗疗效的预测价值及临床应用中相关问题。

## Abstract (en)

The morbidity and mortality of lung cancer is the first in the world, immunotherapy has become a important treatment strategy in addition to chemotherapy, radiotherapy and targeted therapy. In recent years, the US Food and Drug Administration (FDA) has successively approved immunological checkpoint inhibitors as standard programs for non-small cell lung cancer (NSCLC) in second-line or first-line treatment. The National Comprehensive Cancer Network (NCCN) also recommends immunological checkpoint inhibitors as the standard treatment for small cell lung cancer (SCLC). Now, the treatment for lung cancer has entered the era of precision treatment, it is very important to select effective and reliable biomarker for the dominant populations of lung cancer to receive immunotherapy. A large number of researchs indicated that tumor mutation burden (TMB) may be an independent predicted biomarker for immunotherapy, but with limitations. This article reviewed the predictive value of TMB and its limitations in the field of immunotherapy for lung cancer.

肺癌的发病率和死亡率居全球首位，2018年全球将有肿瘤新发病例1, 810万和肿瘤死亡病例960万，其中肺癌占总病例数11.6%，占癌症总死亡人数18.4%^[1]^。晚期非小细胞肺癌（non-small cell lung cancer, NSCLC）患者1年生存率不足40%^[2]^，对于驱动基因阴性的晚期NSCLC患者，仍然缺乏有效的治疗方法。小细胞肺癌（small cell lung cancer, SCLC）约占10%-15%，化疗后进展的患者几乎没有更有效的治疗选择，通常预后较差。

近年来，针对程序性死亡受体1（programmed death 1, PD-1）/配体（PD-1 ligand, PD-L1）免疫检查点抑制剂的治疗发展迅猛，开启了晚期NSCLC治疗的新篇章，但是抗PD-1/PD-L1单药的NSCLC应答率仅为25%^[3]^，因此寻找合适的生物标记物（biomarker）以筛选优势人群，进而提高NSCLC免疫治疗疗效是目前肺癌免疫治疗的重中之重。肿瘤突变负荷（tumor mutation burden, TMB）是近年来在多种肿瘤免疫治疗中发现的可用于预测免疫治疗疗效的独立生物标记物，高TMB表达者对免疫检查点抑制剂治疗临床获益更大^[4]^。但是对于TMB在NSCLC中的免疫疗效预测价值尚存在许多争议，因此本文对TMB在针对PD-1/PD-L1免疫检查点抑制剂的NSCLC免疫治疗疗效的预测价值进行总结。

## 1 免疫检查点抑制剂的作用机制

肿瘤免疫编辑包括免疫监视、免疫平衡和免疫逃逸三个阶段，其中免疫逃逸在肿瘤的发生发展中发挥着最为重要的作用。在抗肿瘤免疫反应中，T细胞介导的细胞免疫反应是最为主要的，只有初始T细胞活化后才能起到抗肿瘤效应。因此免疫治疗最为重要的是解除T细胞活化的抑制信号通路。这种抑制信号通路被称为免疫检查点（immune checkpoints），以细胞毒T淋巴细胞相关抗原4（CTLA-4通路）和抗PD-1及其配体PD-L1为代表。另外IDO、TIM-3、OX40、LAG-3、CD47等作为新的检查点抑制或激活分子也逐渐被发现^[5, 6]^。针对肿瘤细胞的免疫识别及免疫应答相关逃逸机制，科学家们研发出相关的肿瘤免疫治疗药物，包括抗CTLA-4单抗（Ipilimumab、Tremelimumab），通过解除T细胞活化抑制信号，从而恢复T细胞的活化及识别能力；抗PD-1/PD-L1单抗（Nivolumab、Pembrolizumab、Atezolizumab、Durvalumab），通过促进活化T细胞的增殖，激活肿瘤抗原特异性细胞毒性T细胞的杀伤功能来增强免疫反应，从而起到杀伤肿瘤细胞的作用，而这也正是目前免疫检查点抑制剂治疗肿瘤的主要作用原理。

研究发现：虽然NSCLC患者CD8^+^T细胞存在PD-1的过表达，但CD8^+^T细胞却存在PD-1高表达与免疫功能受损的双重状态，表现为细胞因子生成能力下降及和细胞增殖能力减低^[7]^。另一方面，在许多恶性肿瘤（包括NSCLC）中，肿瘤细胞中PD-L1高表达与肿瘤细胞的低分化和预后差有显著的相关性^[8, 9]^。因此，抑制PD-1通路可促进肿瘤特异性T细胞功能的恢复并减少肿瘤诱导的免疫逃逸现象。

## 2 TMB的概念

TMB是指肿瘤细胞基因组中，所评估基因的外显子编码区每兆碱基中发生置换和插入/缺失突变的总数。一方面驱动基因突变可以导致肿瘤的发生，另一方面大量的体细胞突变可以产生新抗原，这些新抗原可以激活CD8^+^的细胞毒性T细胞，从而发挥T细胞介导的抗肿瘤效应^[10]^。因此，当基因变异数目累积增多时，就会产生更多的新抗原，进而被免疫系统识别的可能性就越大。TMB最初是在使用Ipilimumab或Tremelimumab治疗晚期黑色素瘤患者时作为预测疗效的生物标记物^[11]^，结果发现拥有高水平TMB的黑素色瘤和NSCLC患者对PD-1/PD-L1免疫检查点抑制剂疗效往往高于低水平TMB表达的患者^[12]^。既往研究提示：NSCLC中接受抗PD-1/PD-L1免疫检查点抑制剂治疗的患者，与TMB密切相关的非同义突变数量与肿瘤的客观缓解率（objective response rate, ORR）、持续临床获益时间及无进展生存期（progression-free survival, PFS）的改善均呈正相关^[13]^。

## 3 TMB作为生物标记物在肺癌免疫治疗相关的研究进展

基于CheckMate 017^[14]^/057^[15]^两项研究结果，PD-1抑制剂Nivolumab被美国FDA批准用于治疗NSCLC铂类为基础化疗后进展的晚期鳞癌和非鳞癌。2018年2月，*Annals of Oncology*杂志发表了这两项研究3年OS数据汇总分析（随访40.3个月），不论是鳞癌还是非鳞癌，Nivolumab组总生存期（overall survival, OS）较多西他赛组明显延长（HR=0.70, 95%CI: 0.61-0.81），其存在持续的生存获益^[16]^。

基于CheckMate 026/227研究，今年10月NCCN发布了NSCLC指南2019年第1版，指南中指出TMB用于识别适合接受“Nivolumab+Ipilimumab”免疫联合治疗和“Nivolumab”单药治疗的肺癌患者。CheckMate 026^[17]^是首个用于分析TMB与PD-1抑制剂临床获益相关性的Ⅲ期随机对照研究，TMB高表达患者中，Nivolumab组应答率高于化疗组，PFS也显著延长，提示高TMB与免疫治疗疗效呈正相关。

CheckMate 227^[18]^是免疫联合方案对比化疗用于初治晚期或复发NSCLC的首个Ⅲ期临床研究，并且将高TMB患者的PFS作为共同主要终点进行评估。在PD-L1 < 1%的患者中，TMB < 10个突变/Mb亚组中，Nivolumab联合化疗与单纯化疗组进行分析发现两组在ORR、PFS上均无统计学差异；而在TMB≥10个突变/Mb亚组中，Nivolumab联合化疗组的ORR和中位无进展生存期（median PFS, mPFS）均优于单纯化疗组（60.5% *vs* 20.8%，6.2个月*vs* 5.3个月），提示TMB未来可能成为免疫联合化疗方案中筛选优势人群的生物标记物。同时这项研究表明在整体人群中免疫联合组PFS相比化疗并未显现优势（4.9个月*vs* 5.5个月）情况下，在TMB≥10个突变/Mb组患者中，免疫联合组PFS显著优于单纯化疗组，mPFS分别为7.2个月和5.5个月（HR=0.58, *P*=0.000, 2）。而且在高TMB人群中，无论PD-L1表达如何，免疫联合组PFS均优于化疗组。而在低TMB组，PFS分别为3.2个月和5.5个月，无统计学意义。基于以上数据的公布，表明TMB可以作为生物标记物来选择免疫治疗的优势人群，并指出TMB≥10个突变/Mb是筛选最佳获益人群的临界阈值（cutoff值）。

TMB作为生物标记物在SCLC的免疫治疗中仍在探索阶段，SCLC与吸烟密切相关，体细胞突变负荷高，理论上产生的新抗原较多，是免疫治疗的理想肿瘤类型^[19-21]^。CheckMate 032^[22]^研究首次将TMB用于SCLC免疫治疗的疗效预测，结果显示：不论是Nivolumab+Ipilimumab还是Nivolumab单药治疗复发的高TMB小细胞肺癌患者疗效均显著增高，接受双药联合治疗的高TMB患者，ORR为46%，而中低TMB组分别为16%和22%。而接受nivolumab单药治疗的TMB高中低组ORR分别为21%、7%和5%。更出乎意料的是，接受两药联合治疗的高TMB组患者1年OS率为62.4%，而中低组分别为19.6%和23.4%，接受Nivolumab单药的高TMB组患者1年OS率为35.2%，中低组分别为26.0%和22.1%。基于此研究，NCCN指南2017年第1版将Nivolumab±Ipilimumab作为复发SCLC二线治疗的推荐方案之一。

Atezolizumab是针对PD-L1的人源化IgG1单克隆抗体，已被FDA批准用于二线及以上的晚期NSCLC治疗。OAK和POPLAR系列研究^[23]^应用血液中的循环肿瘤DNA（ctDNA）评估血液肿瘤突变负荷（bTMB），并分析bTMB水平与Atezolizumab疗效相关性。结果显示：bTMB≥10个、≥16个和≥20个突变/Mb时均可见PFS和OS获益，但是bTMB≥16个突变的预测水平最佳（PFS HR=0.57，OS HR=0.56），并将此数值作为OAK Ⅲ期研究的cutoff值进行分析。在OAK研究中，bTMB≥16组的患者中，显示Atezolizumab组对比化疗组PFS和OS均显著获益（PFS HR=0.65，OS HR=0.64；mOS 13.5个月*vs* 6.8个月），也进一步证实了bTMB≥16个突变是晚期NSCLC患者的一个可预测治疗疗效的有效界值。另外，如果将bTMB与肿瘤组织TMB（tTMB）进行对比，发现来源于同一个患者的tTMB和治疗前的bTMB呈显著正相关（rs=0.64, 95%CI: 0.56-0.71），阳性率为64%（95%CI: 54-74），特异性为88%（95%CI: 83-92）。B-F1RST是首个前瞻性评估b-TMB作为Atezolizumab一线治疗NSCLC疗效预测标志物的临床研究，采用bTMB≥16个突变作为该研究的cutoff值，研究显示，高bTMB组患者无论是PFS、OS还是ORR，均较低bTMB组患者有明显优势。上述结果也更进一步支持了正在进行的Ⅲ期研究BFAST以bTMB作为标志物来筛选入组人群。

## 4 影响TMB表达的相关因素

TMB表达水平可能与多种因素有关，例如微卫星不稳定（microsatellite instability, MSI-H）及某些驱动基因的存在等。Rizvi等^[13]^对Pembrolizumab治疗晚期NSCLC患者研究时发现治疗疗效与吸烟、新抗原负荷的高表达、DNA修复通路突变（POLD1、POLE和MSH2）密切相关，同时这些因素也都与高TMB相关，因此可以通过TMB来评估治疗疗效。Pembrolizumab被美国FDA批准用于治疗高度微卫星不稳定（MSI-H）或错配修复基因缺陷（dMMR）的晚期进展患者，这也是FDA首次不以肿瘤类型而仅依据生物标记物选择治疗决策的突破。Chalmers等^[24]^对10万例病例研究发现，在MSI-H患者中，97%患者TMB≥10个突变/Mb，83%患者≥20个突变/Mb，相反的是，只有16%高TMB患者具有MSI-H。这两种表型的共同出现高度依赖于肿瘤类型，在胃肠道肿瘤中比如胃腺癌、十二指肠腺癌、小肠腺癌中，MSH-H和高TMB往往共同出现；而在黑素瘤、鳞状细胞癌和肺癌中，高TMB较为常见，而MSI-H罕见。Chalmers等^[24]^也发现DNA错配修复通路基因（*MSH2,* *MSH6,* *MLH1,* *PMS2*）、DNA聚合酶（POLE）、*TOP2A*和*TP53BP1*突变与高TMB相关。既往研究发现某些驱动基因可以影响PD-L1的表达，如*EGFR*、*KRAS*和*ALK*^[25]^。突变的*EGFR*可以上调PD-L1的表达和抑制肿瘤浸润淋巴细胞的激活，但是拥有*EGFR*突变的患者比野生型患者在接受免疫治疗时预后差，也许这与研究中发现的*EGFR*突变可以导致低TMB和肿瘤特异性免疫应答受损有关^[26]^。Coelho等^[27]^发现*KRAS*与*TP53*共同突变时往会伴随着TMB增高。McGranahan等^[28]^研究发现约40%NSCLC存在*HLA*基因杂合子缺失，并且拥有*HLA*基因杂合子缺失的患者绝大部分拥有高TMB。

## 5 TMB在临床应用的局限性

肿瘤免疫原性是肿瘤免疫启动的基础，继PD-L1之后，TMB逐步成为预测免疫检查点治疗疗效的潜在标志物，目前已从临床研究逐步走向临床应用。但是，TMB作为预测标志物还有很多不足之处：（1）TMB的检测由于依赖基因测序，成本远高于依靠免疫组化的PD-L1检测；（2）不同平台的检测标准不统一；（3）年龄对TMB表达水平影响较大，并且两者之间的关系因疾病类型的不同而有所差异^[24]^；（4）TMB的数量不能足以准确预测免疫疗效，必须有能够产生激发免疫反应的免疫性抗原的基因突变；（5）不同类型肿瘤TMB水平差异较大^[24]^。起初TMB的检测是基于WES，但由于其价格昂贵限制了临床应用，所以目前逐渐兴起多基因大Panel进行检测，但是目前多家基因检测公司都形成了自己的TMB检测，并且每个公司的Panel所涵盖的基因不同，设定的cutoff值不同，所以TMB的检测无法标准化，从而无法广泛的应用于临床。Chalmers等^[24]^发现TMB的高低与年龄具有明显的相关性，10岁时中位TMB值为1.67个突变/Mb，88岁时为4.50个突变/Mb，一个符合数据的线性模型预测10岁-90岁之间的TMB差异为2.4倍。但是根据目前报道的临床研究数据发现疗效并不与年龄呈正相关，并且有些研究数据甚至是相反结果，比如CheckMate 057和017研究，CheckMate 057中65岁-75岁亚组，免疫治疗组比化疗组死亡风险降低37%（HR=0.63, 95%CI: 0.45-0.89），而 > 75岁亚组中差异不明显（HR=0.90, 95%CI: 0.43-1.87）；CheckMate 017中65岁-75岁亚组，免疫治疗组比化疗组死亡风险降低49%（HR=0.51, 95%CI: 0.32-0.82），而 > 75岁亚组中免疫治疗组比化疗组死亡风险增加85%（HR=1.85, 95%CI: 0.43-1.87）^[29]^。由此我们可以看出，高TMB并不一定与免疫治疗疗效呈正相关。当然，这结果可能会受很多因素影响，其中的机制可能也比较复杂，需要我们去更一步探索。

## 6 总结与展望

近年来，免疫治疗为肺癌治疗带来了重大变革，从晚期二线到一线，甚至到局部晚期和早期新辅助治疗研究，不论是NSCLC还是SCLC都是一路凯歌。从治疗模式的选择看，不论是免疫单药、免疫联合、与化疗、放疗、抗血管生成靶向药物的联合都显示出了明显的临床获益。但免疫治疗目前仍有很多临床未知，总体有效率较低，因此，还需付诸大量的努力去寻找合适的生物标记物筛选优势人群。目前TMB在肺癌中的预测价值已经在多项临床试验中得到验证，但也有一定程度的局限性。此外，为克服组织样本检测的局限性，bTMB应用逐渐广泛，从POPLAR、OAK研究，再到B-F1RST、BFAST研究，都显示出b-TMB预测免疫治疗疗效的潜力。并且首个验证性文章在*Nature Medicine*杂志已发表。b-TMB是相对无创的检测，外周血标本更容易获得，异质性更小，能够简便的实现动态监测，因此这也是一个非常有前景的研究方向。此外还有更多可探索性免疫标志物，如TCR克隆性、可溶性PD-L1、血清蛋白标记、HLA表型、肠道菌群等，为克服单一生物标记物的局限性，多种联合应用与动态监测将会为未来临床应用带来更大价值。
